# Mendelian randomisation of eosinophils and other cell types in relation to lung function and disease

**DOI:** 10.1136/thoraxjnl-2021-217993

**Published:** 2022-05-10

**Authors:** Anna Guyatt, Catherine John, Alexander T Williams, Nick Shrine, Nicola F Reeve, SpiroMeta consortium, Ian Sayers, Ian Hall, Louise V Wain, Nuala Sheehan, Frank Dudbridge, Martin D Tobin

**Affiliations:** 1 Department of Health Sciences, University of Leicester, Leicester, UK; 2 Division of Respiratory Medicine, University of Nottingham, Nottingham, UK; 3 NIHR Nottingham Biomedical Research Centre, University of Nottingham, Nottingham, UK; 4 NIHR Leicester Biomedical Research Centre, University of Leicester, Leicester, UK

**Keywords:** Asthma Epidemiology, Asthma Genetics, Asthma Mechanisms, COPD epidemiology, COPD exacerbations mechanisms, Eosinophil Biology, Respiratory Infection

## Abstract

**Rationale:**

Eosinophils are associated with airway inflammation in respiratory disease. Eosinophil production and survival is controlled partly by interleukin-5: anti-interleukin-5 agents reduce asthma and response correlates with baseline eosinophil counts. However, whether raised eosinophils are causally related to chronic obstructive pulmonary disease (COPD) and other respiratory phenotypes is not well understood.

**Objectives:**

We investigated causality between eosinophils and: lung function, acute exacerbations of COPD, asthma-COPD overlap (ACO), moderate-to-severe asthma and respiratory infections.

**Methods:**

We performed Mendelian randomisation (MR) using 151 variants from genome-wide association studies of blood eosinophils in UK Biobank/INTERVAL, and respiratory traits in UK Biobank/SpiroMeta, using methods relying on different assumptions for validity. We performed multivariable analyses using eight cell types where there was possible evidence of causation by eosinophils.

**Measurements and main results:**

Causal estimates derived from individual variants were highly heterogeneous, which may arise from pleiotropy. The average effect of raising eosinophils was to increase risk of ACO (weighted median OR per SD eosinophils, 1.44 (95%CI 1.19 to 1.74)), and moderate-severe asthma (weighted median OR 1.50 (95%CI 1.23 to 1.83)), and to reduce forced expiratory volume in 1 s (FEV_1_)/forced vital capacity (FVC) and FEV_1_ (weighted median estimator, SD FEV_1_/FVC: −0.054 (95% CI −0.078 to −0.029), effect only prominent in individuals with asthma).

**Conclusions:**

Broad consistency across MR methods may suggest causation by eosinophils (although of uncertain magnitude), yet heterogeneity necessitates caution: other important mechanisms may be responsible for the impairment of respiratory health by these eosinophil-raising variants. These results could suggest that anti-IL5 agents (designed to lower eosinophils) may be valuable in treating other respiratory conditions, including people with overlapping features of asthma and COPD.

**Key messages**

**What is already known on this topic?**

- Blood eosinophil counts are predictive of response to anti-interleukin-5 (IL5) drugs used to treat asthma. However, the causal nature of the relationship between eosinophils and a broad range of respiratory traits related to asthma and chronic obstructive pulmonary disease (COPD) is not fully understood.

**What this study adds?**

- In this Mendelian randomisation study, while the average effect of raising eosinophils was to increase risk of asthma-COPD overlap and asthma, and worsen forced expiratory volume in 1 s (FEV_1_) and FEV_1_/forced vital capacity in individuals with asthma, heterogeneity of individual causal estimates means caution is needed when interpreting these results causally, as these results could also be consistent with eosinophil-raising genetic variants impairing respiratory health via other causal pathways.

**How this study might affect research, practice or policy?**

- These results could suggest that anti-IL5 agents (designed to lower eosinophils) may be valuable in treating other respiratory conditions, including people overlapping features of both asthma and COPD. Future work should seek to explore other potential mechanisms besides eosinophils by which anti-IL5 agents may improve respiratory health, to inform whether the clinical indications for anti-IL5 agents or biomarkers for stratifying their use could be extended.

## Introduction

Eosinophils are proinflammatory granulocytes associated with symptom severity and exacerbation frequency in asthma and chronic obstructive pulmonary disease (COPD).^1–3^ The degree of eosinophilia (raised eosinophils) in these obstructive lung diseases varies: while eosinophil inflammation due to allergic sensitisation has been considered characteristic of asthma, not all patients with asthma have eosinophilia.^1 4^ Moreover, while airway inflammation in COPD is typically mediated by neutrophils, some individuals with COPD have raised eosinophils.^1 5^

The production and survival of eosinophils is partly regulated by interleukin-5 (IL-5), and anti-IL5 therapies (eg, mepolizumab, reslizumab, and the anti-IL5Rα agent, benralizumab) are now licensed in many countries for the treatment of severe eosinophilic asthma.^6–12^ The decision to treat asthma with these drugs is currently based on blood eosinophil count, among other factors,^1^ since post-hoc analyses of clinical trials stratified by eosinophil levels have shown increased efficacy of mepolizumab for treating severe asthma in those with higher baseline eosinophils.^2^ Results from Mendelian randomisation (MR) analyses have also provided evidence for a role of eosinophils in asthma (estimated OR 1.70 (95% CI 1.53 to 1.91).^13^ MR analyses use genetic variants as instrumental variables (IVs) to investigate causality between exposure and outcome, and under certain assumptions may obviate problems with traditional observational epidemiology (eg, reverse causation, confounding), permitting causal inference.

In addition to asthma, blood eosinophils are associated with quantitative lung function in general populations (ie, including individuals without asthma).^14^ However, causality has yet to be established: an inverse relationship between eosinophils and lung health has been suggested, yet a previous MR of lung function (plus another including asthma and COPD) were of small sample size, with imprecise estimates precluding confident inference.^15 16^ Moreover, causality of eosinophils on other respiratory phenotypes, for example, asthma-COPD overlap (ACO), and respiratory infections are yet to be investigated. COPD is diagnosed by spirometry if the ratio of the forced expiratory volume in 1 s (FEV_1_) to forced vital capacity (FVC), FEV_1_/FVC, is <0.7, with airflow obstruction graded by predicted FEV_1_. Therefore, studying eosinophils as determinants of quantitative lung function is a powerful way of understanding their role in the development of fixed airflow obstruction such as in COPD.^17 18^ Investigating causality between eosinophils and fixed airflow obstruction is pertinent given interest in the potential use of mepolizumab in COPD^9–12^; evidence for causality of eosinophils in a wider range of respiratory phenotypes could suggest that anti-IL5 agents (designed to lower eosinophils) might be helpful in conditions beyond asthma.

We undertook two-sample MR analyses using summary-level genome-wide association study (GWAS) data to assess causality between eosinophils and conditions encompassing fixed and reversible airflow obstruction, using genetic variants associated with blood eosinophils as IVs.^13^ We investigated causality of eosinophils on three quantitative lung function spirometry traits, and four clinical phenotypes (moderate-to-severe asthma, acute exacerbations of COPD (AECOPD), ACO and respiratory infections). We used MR approaches relying on different assumptions for validity, and followed up traits showing evidence of possible causality to assess evidence that the IVs affected lung function via eosinophil counts and not via other blood cell types. Overall, our aim was to provide a comprehensive assessment of the causal role of blood eosinophil counts in relation to respiratory health and disease.

## Methods

**Figure 1:**
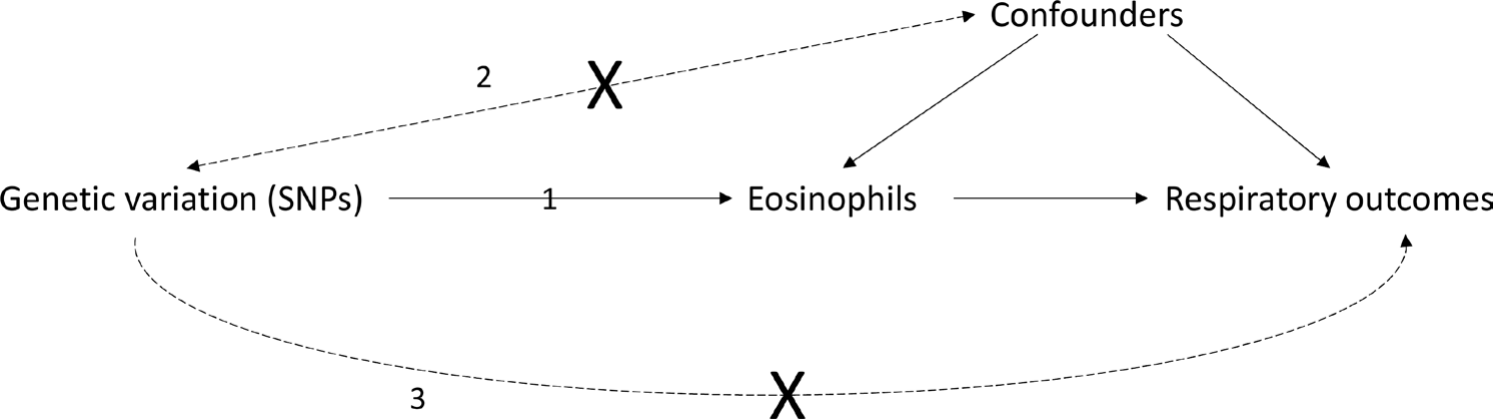
Mendelian randomisation (MR): core assumptions Mendelian randomisation may be used to test for causality between an exposure (eg, eosinophils) and outcome (eg, a respiratory outcome such as FEV_1_/FVC), if the following core assumptions hold (see 1–3 on the figure): (1) the genetic variation (single nucleotide polymorphisms in this work) used as instrumental variables are associated with the exposure of interest; the genetic variants are not associated with unobserved confounders of the exposure-outcome association (straight dashed arrow). Genetic variants are allocated randomly at conception (Mendel’s law of independent assortment) and so typically should not be associated with these confounding variables; association between the genetic variants and the outcome is via the exposure, and not via an alternate pathway (ie, there is no ‘horizontal pleiotropy’, see curved dashed arrow). While difficult to verify, reassurance that this assumption holds can be provided using biological knowledge of how the SNP functions, and by checking whether multiple MR methods, each relying on different assumptions for validity, give consistent results (known as triangulation).^20^ FEV_1_, forced expiratory volume in 1 s, FVC, forced vital capacity; SNP, single-nucleotide polymorphisms.

We assessed causality between eosinophils and other blood cell counts in relation to respiratory outcomes using MR.^19 20^ MR involves using genetic variants (here single-nucleotide polymorphisms, SNPs), as IVs for an exposure of interest, in this case eosinophil counts, by comparing the magnitude of the effect of the SNPs on the outcome to the effect of the SNPs on the exposure.^19 20^ All analyses reported are two-sample MR analyses, since SNP–exposure and SNP–outcome associations were extracted from different (yet overlapping^21^) samples. Core MR assumptions for inferring causality between are that: (1) the genetic variants are associated with the exposure of interest; (2) there are no unmeasured confounders of the associations between genetic variants and outcome; and (3) the genetic variants affect the outcome only via the exposure of interest (figure 1).^19^ Additional assumptions for accurate point estimation of effect sizes are discussed in online supplemental file 1, and elsewhere.^22^

10.1136/thoraxjnl-2021-217993.supp1 Supplementary data

All GWAS datasets analysed included UK Biobank, a prospective cohort study including spirometry, biological assays, questionnaire data, and linked healthcare records, and 450 000 participants with genotype data.^23^ Other studies were incorporated where available, and all GWAS data were from individuals of European ancestry. Datasets are summarised below, and descriptions of covariate adjustments, and exposure-outcome GWAS overlap are given in the extended methods (online supplemental file 1).

### Exposure GWAS data sets (blood cell parameters)

We used summary-level data from eight published GWASs of blood cell counts^13^ in the initial release of UK Biobank genetic data (N up to 132,959, that is, around 30% of participants with genotype data), plus the INTERVAL study (N up to 40 521)).^13^ GWASs were of blood eosinophils, basophils, neutrophils, monocytes, lymphocytes, platelets, red blood cells and reticulocytes, with adjustments for technical and seasonal covariates, plus age, menopausal status, height, weight, smoking and alcohol (online supplemental file 1).

### Outcome GWAS data sets (respiratory outcomes)

See also online supplemental file 1.

#### Quantitative lung function GWASs

We used published summary-level data from three GWAS of FEV_1_, FVC and FEV_1_/FVC, in UK Biobank (n=3 21 047) and the SpiroMeta consortium (n=79 055).^18^ Prior to GWAS, traits were preadjusted for age, age^2^, sex, height, smoking status and other covariates as appropriate, for example, ancestry principal components. Residuals were inverse-normal rank transformed.

#### Clinical outcome GWAS

##### Moderate-to-severe asthma

We used a published GWAS of moderate-to-severe asthma within the Genetics of Asthma Severity and Phenotypes initiative, the U-BIOPRED asthma cohort, and UK Biobank.^24^ Cases (n=5135) were taking asthma medication, and met criteria for moderate-to-severe asthma (British Thoracic Society 2014 guidelines). Controls (n=25 675) excluded those with a doctor diagnosis of asthma, rhinitis, eczema, allergy, emphysema, or chronic bronchitis, or missing medication data. Analyses were adjusted for 10 ancestry principal components.

##### Acute exacerbations of COPD

We defined AECOPD in UK Biobank; the eligible sample was restricted to individuals with FEV_1_/FVC<0.7. Exacerbation cases (n=2771) had an ICD-10 code for AECOPD or a lower respiratory tract infection in Hospital Episode Statistics data (online supplemental table 1). Controls (n=42 052) had FEV_1_/FVC<0.7, without an AECOPD code. Associations were adjusted for age (at recruitment), age^2^, sex, smoking status (ever/never), genotyping array and 10 principal components.

10.1136/thoraxjnl-2021-217993.supp3 Supplementary data

##### Asthma-COPD overlap

We defined ACO in UK Biobank (N=8068) as individuals self-reporting a doctor diagnosis of asthma, with FEV_1_/FVC<0.7 and FEV_1_ <80% predicted at any study visit. Controls (N=40 360) were selected in approximately a 5:1 ratio, from participants reporting no asthma or COPD, (FEV_1_ >80% predicted, FEV_1_/FVC>0.7). Associations were adjusted for age (at recruitment), sex, smoking status and 10 principal components.^25^

##### Respiratory infections

We defined respiratory tract infections requiring hospital admission in UK Biobank, using the ICD-10 codes in online supplemental table 2. Cases had ≥1 admission for respiratory infections (N=19 459). Controls had no admissions for respiratory infections and were selected in approximately a 5:1 ratio (N=101 438). Associations were adjusted for age (at recruitment), age^2^, sex, smoking status, genotyping array, and 10 principal components.^26^

### Statistical methods

#### Univariable MR of eosinophils and respiratory traits and diseases

We performed separate MR analyses of eosinophils on three quantitative lung function traits (FEV_1_, FVC, FEV_1_/FVC); and four clinical phenotypes (asthma, AECOPD, ACO, respiratory infections) using genetic IVs from the work of Astle and colleagues.^13^ Selection of 151 eosinophil IVs and harmonisation of SNP-exposure and SNP-outcome datasets is detailed in the online supplemental file 1. The primary MR analysis used the inverse-variance weighted (IVW) method and a random-effects model, which will return a valid causal estimate provided that the average pleiotropic effect is zero. We investigated the ‘no pleiotropy’ assumption using MR-Egger regression,^27^ the weighted median estimator^28^ and MR-PRESSO^29^ (see online supplemental file 1 for details on assumptions relied on for validity by each method). Further sensitivity analyses: (1) investigated robustness of findings to heterogeneity using MR-PRESSO (for traits with some evidence of causation by eosinophils), (2) restricted to non-UKB FEV_1_/FVC GWAS data, to assess sensitivity to sample overlap and (3) restricted to FEV_1_/FVC GWAS data in UKB, stratifying by asthma status.

#### Multivariable MR analyses of multiple blood cell types and respiratory outcomes

Since SNPs affecting eosinophils also affect other blood cell types,^13^ we used multivariable MR to estimate the influence of multiple cell types on respiratory outcomes, after conditioning on the effects of the SNPs on other cell types. Multivariable MR analyses were performed for respiratory outcomes with evidence of eosinophil causation in the IVW MR analyses above, and with broadly consistent effect estimates in the weighted median and MR-Egger analyses. We also performed an analysis of FEV_1_/FVC in UKB (stratifying by asthma status).

There were 1166 SNPs associated with at least one of eight blood traits reported by Astle and colleagues^13^ at a genome-wide threshold. These SNPs were LD clumped, and effect sizes extracted from each blood cell GWAS, and each outcome GWAS. Effects for 318 clumped SNPs were harmonised, that is, so effect sizes for SNP-exposure and SNP-outcome effects corresponded to the same allele (online supplemental table 3, online supplemental file 1). Conditional F-statistics were estimated using the strength_mvmr() function of the ‘MVMR’ R package.^30^

For IVW multivariable MR analyses, we used the mv_multiple() function of the ‘TwoSampleMR’ R package.^31–33^ This analysis aimed to further investigate the possibility of horizontal pleiotropy affecting the results of the univariable eosinophil MR; and to establish whether other blood cell types besides eosinophils could affect the respiratory outcomes studied.

Sensitivity MVMR methods (online supplemental file 1) included: (1) use of an MVMR method more robust to pleiotropy in the presence of weak instruments (using the qhet_mvmr() function of the ‘MVMR’ R package,^30^—standard errors calculated by a jack-knife approach) and (2) recalculation of IVW MVMR estimates after removal of SNPs contributing most to heterogeneity (SNPs identified using the pleiotropy_mvmr() function).

## Results

### Univariable MR analyses of eosinophils and respiratory outcomes

**Figure 2:**
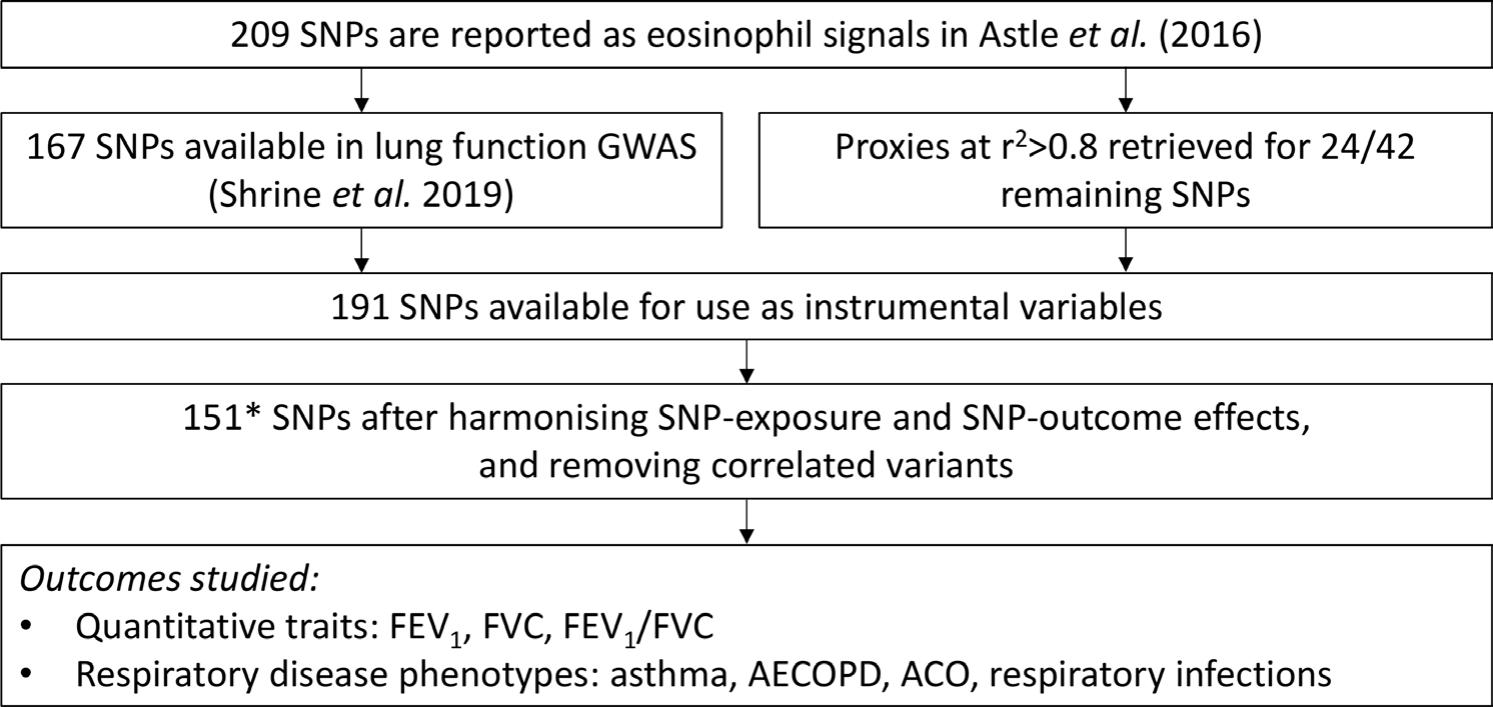
Selection of SNPs for univariable MR analyses of eosinophils and respiratory outcomes flow chart describing the analysis workflow for initial MR analyses of eosinophils. Of 209 SNPs associated with eosinophil count, 167 were available in lung function GWASs (missingness is due to some SpiroMeta studies not being imputed to the HRC panel).^18^ LD proxies at R^2^ >0.8 were retrieved for 24/42 missing variants. Of the resulting 191 SNPs, 188 were successfully harmonised between the SNP-eosinophil and SNP-lung function data sets, and 151* remained after LD clumping at an R^2^ threshold of 0.01. These 151 SNPs were used in analyses. *One SNP, rs9974367, was missing in the moderate-severe asthma GWAS. AECOPD, acute exacerbation of COPD; ACO, asthma COPD overlap; COPD, chronic obstructive pulmonary disease; FEV_1_, forced expiratory volume in 1 s, FVC, forced vital capacity; GWAS, genome-wide association study; MR, Mendelian randomisation; SNPs, single-nucleotide polymorphisms.

There were 151 SNPs available for the univariable MR analyses of three quantitative traits (FEV_1_, FVC and FEV_1_/FVC), and four respiratory disease phenotypes (moderate-to-severe asthma, AECOPD, ACO and respiratory infections). Details of SNP selection are described in figure 2.

Results are presented in figure 2. Among the quantitative traits, there was evidence for an effect of eosinophils on FEV_1_/FVC (SD change in FEV_1_/FVC per SD eosinophils, IVW estimate=−0.049 (95% CI −0.079 to–0.020)), with a smaller effect on FEV_1_ (IVW estimate=−0.028 (95% CI −0.054 to –0.003)). However, there was substantial heterogeneity of SNP-specific causal estimates, as evidenced by the large values of Cochran’s Q statistic, suggesting that core MR assumptions were violated for at least some SNPs. Scatterplots of SNP-outcome against SNP-exposure effects are given in online supplemental figure 1).

10.1136/thoraxjnl-2021-217993.supp2 Supplementary data

**Figure 3:**
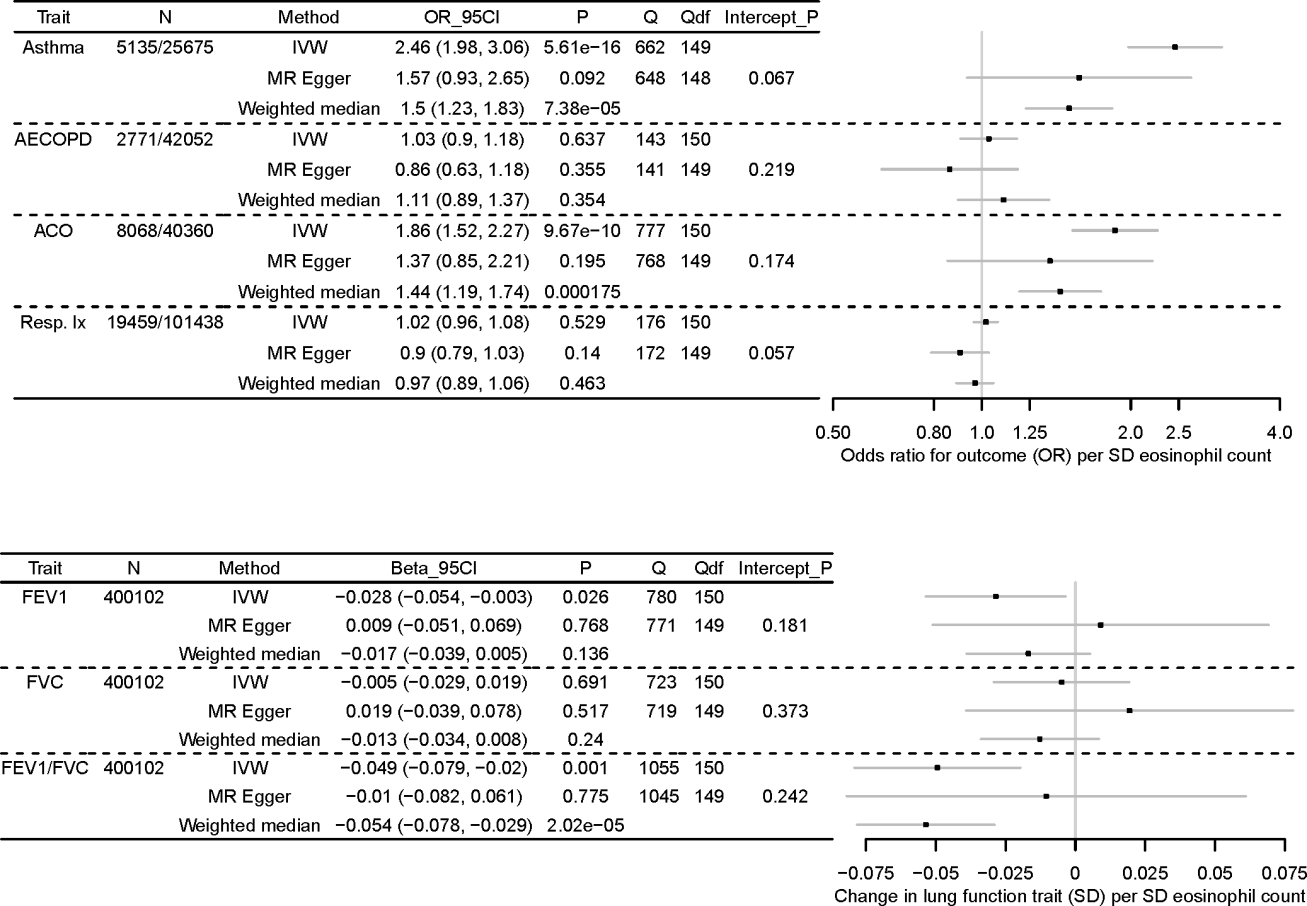
MR analyses of eosinophils (exposure) on three quantitative lung function traits (top) and four respiratory disease phenotypes (bottom), using 151 eosinophil-associated SNPs top: results of MR analyses of eosinophil counts (exposure) on three quantitative lung function traits (outcome), FEV_1_, FVC and FEV_1_/FVC. A forest plot of three estimates for each traits is shown (IVW, MR Egger, weighted median), along with the maximum sample size in the outcome GWAS (N), the effect size in SD change in outcome trait per SD increase eosinophil count, and 95% CI, values for Cochran’s Q statistic (Q) and the associated df (Q_df), and the p value for the MR Egger intercept (Intercept_P). Boxes of the forest plot represent effect sizes, whiskers are 95% CIs. Bottom: results of MR analyses of eosinophil counts (exposure) on four respiratory disease phenotypes (outcome), moderate-to-severe asthma, acute exacerbations of COPD (AECOPD), asthma-COPD overlap (ACO), and respiratory infection (Resp. IX). A forest plot of three estimates for each traits is shown (IVW, MR Egger, weighted median), along with sample size in the outcome GWAS for cases and controls, respectively (N), the effect size as OR per SD eosinophil count, and 95% CI, values for Cochran’s Q statistic (Q) and the associated df (Q_df), and the p value for the Mr Egger intercept (Intercept_P). Boxes of the forest plot represent ORs, whiskers are 95% CIs. Nb only 150/151 of the eosinophil SNPs were available in the moderate-to-severe asthma GWAS. COPD, chronic obstructive pulmonary disease; FEV_1_, forced expiratory volume in 1 s, FVC, forced vital capacity; GWAS, genome-wide association study; IVW, inverse-variance weighted; MR, Mendelian randomisation; SNPs, single-nucleotide polymorphisms.

Among the respiratory disease phenotypes (figure 3), there was evidence for an effect of eosinophils on asthma (OR per SD eosinophil count, IVW method=2.46 (95% CI 1.98 to 3.06)), and ACO (IVW OR=1.86 (95% CI 1.52 to 2.27)). There was substantial heterogeneity of SNP-specific causal estimates for these two traits, and weighted median estimates were of smaller magnitude than IVW estimates (weighted median OR: 1.50 (95% CI 1.23 to 1.83) for asthma, and 1.44 (95% CI 1.19 to 1.74) for ACO). While confidence intervals for the MR Egger estimates were still broad, estimates were generally similar to weighted median estimates. The asthma estimates in particular may have been inflated by overlap between the SNP-exposure and SNP-outcome datasets (see online supplemental file 1). Scatterplots of SNP-outcome against SNP-exposure effects for these outcomes are given in online supplemental figure 2.

There was no evidence of association of eosinophils with AECOPD or respiratory infections. CIs for all three MR methods included the null, and point estimates approached the null. See online supplemental table 4 for results for all models and all traits.

#### Sensitivity analysis to assess further the robustness of findings to heterogeneity, using MR-PRESSO

For FEV_1_, FEV_1_/FVC, ACO and asthma (traits showing strongest evidence of causation), we used MR-PRESSO to identify possible pleiotropic outliers (online supplemental table 5). Results were qualitatively similar to IVW estimates (higher eosinophils consistent with respiratory morbidity), but ACO and asthma effect estimates attenuated after MR-PRESSO outlier correction; MR-PRESSO estimates were most similar to weighted median causal estimates.

#### Sensitivity analysis to assess the effects of sample overlap for quantitative lung function traits

UK Biobank featured in all GWAS datasets used, although the blood cell count GWAS and asthma GWAS included only approximately one third of the UK Biobank genotype data.^13^ We conducted sensitivity analyses to assess for the effect of sample overlap, since we had access to quantitative lung function GWAS data without UK Biobank participants (see online supplemental file 1). Results were generally consistent (SD change in FEV_1_/FVC per SD eosinophil count, IVW estimate=−0.041 (95% CI −0.072 to –0.009); SD change FEV_1_ per SD eosinophil count=−0.043 (95% CI −0.077 to –0.010)) (online supplemental table 6).

#### Sensitivity analysis to assess the effect on FEV_1_/FVC in individuals with and without asthma

The causal effect of eosinophils on FEV_1_/FVC was recalculated using data from UK Biobank, stratifying by asthma status (37 868 cases, 283 179 controls). The effect size was larger in individuals with asthma (IVW −0.083 (95% CI −0.139 to –0.028)) than in those without asthma, in whom there was no effect (IVW −0.013 (95% CI −0.041 to 0.015)). However, confidence intervals for both subgroups overlapped one another (see online supplemental table 7).

### Multivariable MR analyses of blood cell counts and respiratory outcomes

To further explore causality between blood cell parameters and FEV_1_, FEV_1_/FVC, moderate-to-severe asthma and ACO, and to see if other exposures could have accounted for the heterogeneity observed in the previous analyses, we carried out multivariable MR analyses, using eight cell type exposures (eosinophils, basophils, neutrophils, monocytes, lymphocytes, platelets, red blood cells and reticulocytes).

Selection of 318 SNP IVs for multivariable MR is described in online supplemental file 1, online supplemental table 3. SNPs used in the univariable and multivariable MR are listed in online supplemental tables 8 and 9. Briefly, 1166 unique SNPs were associated with at least one of the eight cell types at a genome-wide level in the cell type GWAS, and were available in outcome GWAS. After LD-clumping, 329 SNPs remained, and after harmonising SNP-exposure and SNP-outcome effects, 318 remained (see online supplemental table 3) for conditional F statistics, which were all F>10, except for basophils (F_conditional_=8).

**Figure 4:**
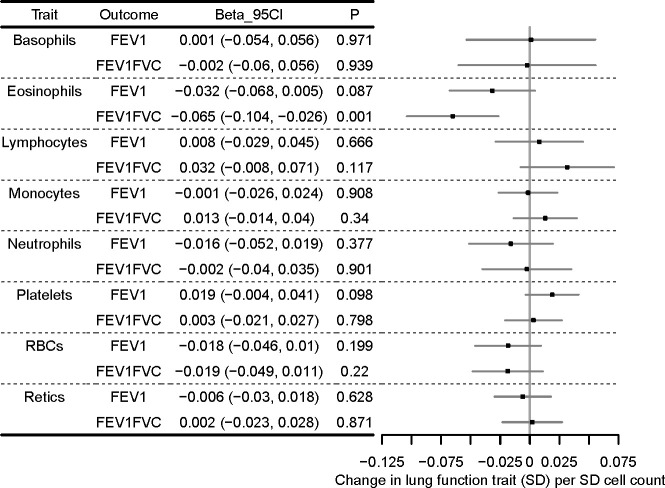
Multivariable MR analyses of eight cell types and forced expiratory volume in 1 s (FEV_1_) and FEV_1_/forced vital capacity (FVC) forest plot showing multivariable MR estimating the causal effect of multiple cell types on quantitative lung function outcomes, after conditioning on the effects of the SNPs on other cell types. Models were run for each of FEV_1_ and the ratio of FEV_1_ to FVC separately, but effect sizes are shown next to one another for comparison. Effect sizes (beta, 95% CI) are in SD change in lung function outcome per SD cell count (adjusted for the effects of other cell types). Points of the forest plot represent effect size estimate; whiskers are 95% CIs. MR, Mendelian randomisation.

Multivariable MR results for FEV_1_ and FEV_1_/FVC are presented in figure 4. Even after conditioning on the effects of the SNPs on other cell types, the average effect of the eosinophil-lowering IVs was to reduce lung function as measured by FEV_1_/FVC (multivariable estimate, SD change in FEV_1_/FVC per SD eosinophils adjusted for other cell types: −0.065 (95% CI −0.104 to –0.026)). The eosinophil point estimate for FEV_1_ (−0.032 (95% CI −0.068 to 0.005)) was consistent with the univariable estimate (figure 3), but CIs for all cell types were consistent with the null. When asthma cases were excluded from SNP-FEV_1_/FVC results, the eosinophil estimate attenuated, and confidence intervals overlapped the null (−0.028 (95% CI −0.069 to 0.013)), consistent with the causal effect of eosinophils on lung function being of greater magnitude in people with a history of asthma (online supplemental figure 3).

**Figure 5:**
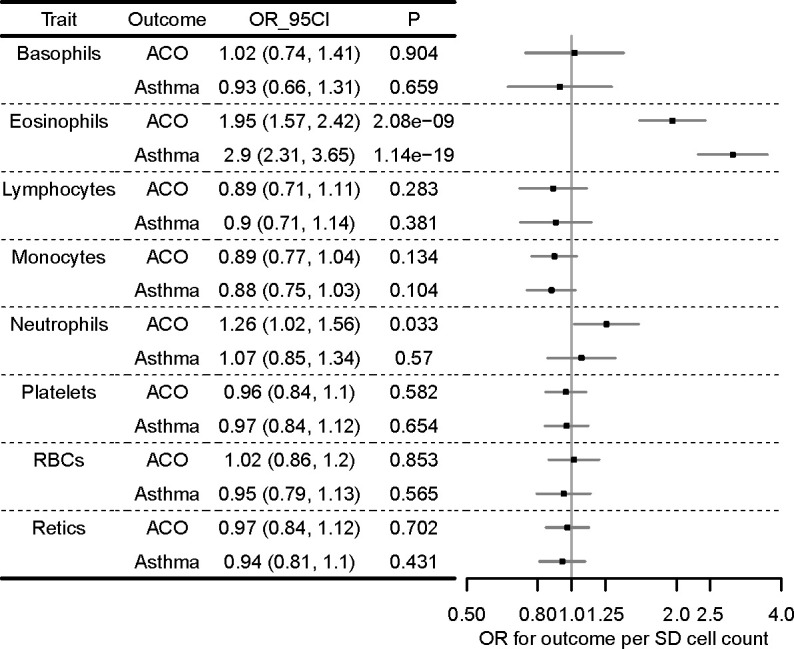
Multivariable MR analyses of eight cell types and two respiratory disease outcomes, ACO and asthma forest plot showing multivariable MR estimating the causal effect of multiple cell types on respiratory disease outcomes, after conditioning on the effects of the SNPs on other cell types. Models were run for each of ACO and asthma separately, but effect sizes are shown next to one another for comparison. ORs (95% CI) are per SD cell count (adjusted for the effects of other cell types). Points of the forest plot represent ORs; whiskers are 95% CIs. ACO, asthma-COPD overlap; MR, Mendelian randomisation; SNP, single-nucleotide polymorphisms.

Results of the multivariable MR analysis for ACO and asthma are presented in figure 5. There was an association of eosinophil count with both ACO (OR 1.95 (95% CI 1.57 to 2.42)) and asthma (OR 2.90 (95% CI 2.31 to 3.65)), after adjusting for the effects of the SNPs on other cell types. Confidence intervals for other cell type estimates were consistent with the null, with the exception of neutrophils for ACO. None of the additional seven cell types showed strong evidence of causality.

#### Sensitivity multivariable MR analyses

Sensitivity MVMR analyses (1) used an estimation technique more robust to balanced pleiotropy and (2) repeated IVW MVMR, omitting SNP IVs with the most evidence of heterogeneity. Effect directions of sensitivity analyses and the main MVMR analyses were concordant for FEV_1_, FEV_1_/FVC, ACO, and asthma. However, CIs for FEV_1_ and FEV_1_/FVC were broad, and overlapped the null. For ACO and asthma estimates, there was still evidence of an effect, although attenuated in both analyses (estimates from analysis more robust to pleiotropy; ACO OR 1.57 (95% CI 1.07 to 2.30); asthma OR 2.66 (95% CI 1.65 to 4.33); estimates after omitting the most heterogeneous SNPs: ACO OR 1.51 (95% CI 1.23 to 1.85); asthma OR 2.29 (95% CI 1.84 to 2.86)).

## Discussion

In MR analyses, we found that the average effect of raising eosinophils was to decrease FEV_1_/FVC and FEV_1,_ and to increase ACO and asthma risk, and there was broad consistency across MR methods. However, causal estimates of individual variants were highly heterogeneous, suggesting that caution is needed in concluding causal inference: some IVs may have violated MR assumptions, and other important genetically correlated mechanisms could be responsible for the effect on lung health and disease by the eosinophil-raising variants studied.

To our knowledge, this is the largest MR of eosinophils and lung function, and the first to investigate eosinophils and AECOPD, ACO and respiratory infections. Terminology of ACO has changed over time, yet recognition that asthma and COPD coexist in some patients has not changed,^34^ and this is what our analysis aimed to capture.

A previous two-sample MR of eosinophils and asthma was undertaken by the authors of the GWAS that discovered the eosinophil IVs used; this MR analysis used asthma GWAS data from the GABRIEL study.^13^ We are aware of one other small MR of eosinophils and asthma, COPD, FEV_1_ and FEV_1_/FVC, conducted in the LifeLines cohort (N=13 301, 5 SNPs IVs).^15^ In that study, CIs for causal estimates of eosinophils overlapped the null, although point estimates were consistent with a harmful effect for FEV_1_/FVC, asthma and COPD. We used a larger eosinophil GWAS (N=172 275)^13^ to derive IVs, and found that the average effect of eosinophil-raising IVs was to reduce FEV_1_/FVC, the trait used in COPD diagnosis and FEV_1_, used to grade COPD airflow limitation. However, sensitivity analyses highlighted a larger causal estimate of eosinophils on FEV_1_/FVC among those with asthma, with effect estimates attenuating when excluding this group. These findings may highlight the importance of eosinophils as a marker of impaired lung function and airflow obstruction in people with a history of asthma.

We highlight a need for caution in inferring simple causation between eosinophils and these phenotypes, since high degrees of heterogeneity in our results may arise from pleiotropy. To investigate, we compared MR methods relying on differing assumptions for validity (Methods section). Attenuation of some results when using the MR-Egger, weighted median, and MR-PRESSO approaches suggests that some SNP IVs are associated with asthma and ACO via pathways other than eosinophils, which is a known challenge in MR studies (see also Methods section).

Since many of the eosinophil SNP IVs are also associated with other cell counts,^13^ we performed multivariable MR to estimate the influence of multiple cell types simultaneously, after conditioning on the effects of the SNPs on other cell types. While we did not find substantial evidence for a harmful effect of neutrophils on asthma, nor a protective effect of monocytes and lymphocytes, as reported previously,^13^ effect directions in our IVW multivariable MR were consistent with the previous study for neutrophils, monocytes and lymphocytes. We observed a larger effect of eosinophils on asthma than reported previously: this could be because our SNP-outcome dataset was of moderate-to-severe asthma (which has a higher point estimate of genetic correlation with eosinophils), but also, around half of the cases and the majority of controls were also included in the exposure GWAS, which may make this analysis closer to a one-sample MR, and inflate causal effect estimates. Notably, effect sizes partly attenuated in sensitivity analyses which may be more robust to heterogeneity. The MR estimates from multivariable analyses, and the MR-Egger regression and weighted median univariable analyses were consistent with the previous estimate reported for asthma in multivariable analysis by Astle *et al*.^13^ Nevertheless, these limitations may preclude precise estimation of effect sizes, and our results may be more useful in terms of assessing whether there is causality between eosinophils and the phenotypes studied, as opposed to providing estimates of the magnitude of any causal effect between phenotypes.

While we did not find strong evidence for causality of eosinophils on AECOPD and respiratory infections, point estimates were consistent with a harmful effect on AECOPD, and may have been limited by power. The effects of anti-IL5 drugs that have been attributed to the reduction of eosinophils have been noted to be smaller in AECOPD compared with asthma.^2 35^

Key strengths are that we used MR methods with differing sensitivities to underlying assumptions. We a large GWAS of eosinophil counts, to provide a comprehensive assessment of the role of blood eosinophils in relation to multiple respiratory health and disease outcomes. Another strength is that we undertook multivariable MR to investigate causality between multiple cell types and the outcomes studied, while controlling for the effects of IVs that may have had pleiotropic effects via other cell types.

We acknowledge several limitations. We did not have post-bronchodilator measures of spirometry. We used GOLD Stage 2–4 COPD (prebronchodilator FEV_1_ <80% predicted) when defining ACO; using the same prebronchodilator spirometry definition of COPD, a positive predictive value of 98% for diagnosis of postbronchodilation-defined COPD has been shown.^36^ Sample overlap between the SNP-eosinophil and SNP-outcome datasets (all included participants from UK Biobank) could bias estimates towards the observational eosinophil-outcome association^21^; we repeated the univariable MR analysis of eosinophils using SNP-lung function results excluding UK Biobank participants, and observed a consistent IVW estimate. Nevertheless, our other analyses (particularly the asthma analysis) could be vulnerable to some non-conservative bias.^19 21^ GWAS analyses of cell counts have, since analysis, been extended to a larger sample across UKB, and future work deriving IVs from this study would be valuable.^37^ UK BiLEVE participants (a subset of UK Biobank selected for extremes of respiratory traits), were overrepresented in Astle *et al*, which used the interim release of UKB data. While correlation between effect sizes from the two GWAS for the 151 IVs used in this analyses were high, the possibility of selection effects remains. Our MR analyses also use genome-wide results adjusted for covariates, and therefore may be susceptible to collider bias.^19 38^ There is also potential bias in the causal estimates for binary outcomes due to non-collapsibility of the OR,^22^ and we did not consider the possibility of non-linear effects. The multivariable analyses may still be vulnerable to pleiotropy via pathways other than the eight cell types studied, so while we cannot strongly assert causality of eosinophils on lung function, neither do we rule it out, as our results are consistent with a causal effect.

At present, treatment with anti-IL5/anti-IL5Rα agents in asthma is initiated according to eosinophil counts and other factors,^8^ yet it is possible that a more proximal factor may be an even better predictor of drug response. Future work could seek therefore to identify whether particular pathways upstream of eosinophil counts might help design better methods for deciding on treatment initiation. In addition, use of suitable IVs for IL5 levels would permit two-step MR analyses, assessing for a mediating effect of eosinophils on the action of anti-IL5 agents in reducing respiratory morbidity.

To conclude, using MR, we found that the average effect of raising eosinophils was to increase risk of ACO and asthma, and to reduce FEV_1_/FVC (the latter association was only prominent in individuals with asthma). Broad consistency across MR methods is suggestive of a causal effect of eosinophils on asthma overall, and in individuals with features of both asthma and fixed airflow obstruction, although of uncertain magnitude. However, given heterogeneity in results derived from individual IVs, which may indicate violation of MR assumptions, we highlight a need for caution, since alternative mechanisms may be responsible for the impairment of respiratory health by these eosinophil-raising variants. These results could suggest that anti-IL5 agents (designed to lower eosinophils) may be of value in a wider range of respiratory traits, including people with features of both asthma and COPD. Future work should seek to explore other potential mechanisms besides eosinophils by which anti-IL5 agents may improve respiratory health.

## Data Availability

Code used in the analysis for this paper is available on request. Summary-level statistics for the IVs used in this paper are available in the relevant published papers, or are available on request where not already published. Summary-level lung function GWAS and blood cell GWAS data are available from https://www.ebi.ac.uk/gwas/.
