# Supplementary material for: Mendelian randomisation of eosinophils and other cell types in relation to lung function and disease
Source: Thorax. 2022 May 10;78(5):496–503. doi: 10.1136/thoraxjnl-2021-217993 (PMC10176352; doi:10.1136/thoraxjnl-2021-217993)
Supplement: Supplementary data [file thoraxjnl-2021-217993supp001.pdf]

Extended Methods

Further details on the published GWAS datasets included in this analysis are described below (see also original publication references, which are given for each study).

See also Supplementary References listed at the end of this document.

Exposure GWAS data sets (blood cell parameters) (Astle *et al.* 2016)[1]

Summary-level data from a GWAS of blood cell count parameters[1] undertaken in the interim release of UK Biobank (UKB) and INTERVAL studies (N=172,275) were downloaded from the EBI GWAS Catalog (https://www.ebi.ac.uk/gwas/). We first used the results for eosinophil counts in MR analyses of lung function and respiratory disease. We then extended our analysis to simultaneously model the causal effects of additional cell types (e.g. counts of basophils, neutrophils, monocytes, lymphocytes, platelets, red blood cells and reticulocytes) in multivariable MR analyses (see 'Multivariable MR analyses').

In UKB, blood samples were collected at the assessment centre visit, and in INTERVAL, blood samples were taken during routine blood donation. Prior to GWAS, the study's authors adjusted the counts for biological and technical laboratory covariates, and GWAS results were provided as SD change in transformed cell count, per risk allele. Adjustments were made for technical and seasonal covariates, as well as age, menopausal status, height, weight, smoking and alcohol. Full details of covariates and transformations are given in Astle *et al.*[1]

Published outcome GWAS data sets (respiratory outcomes)

Quantitative lung function GWASs (Shrine et al., 2019a)[2]

We used published summary-level data from three GWAS of FEV_1, FVC and FEV_1/FVC, undertaken in UK Biobank (N=321,047) and the SpiroMeta consortium (N=79,055).[2] Prior to GWAS, traits were pre-adjusted for age, age^2, sex, height, smoking status and other covariates as appropriate, e.g. ancestry principal components. Residuals were inverse-normal rank transformed. UK Biobank and SpiroMeta results were combined by meta-analysis. GWAS results restricted to the SpiroMeta consortium only were used to assess the effect of sample overlap in sensitivity analyses.

Moderate-to-severe asthma GWAS (Shrine et al., 2019b)[3]

We used a GWAS of moderate-to-severe asthma within the Genetics of Asthma Severity and Phenotypes (GASP) initiative, with additional cases included from the U-BIOPRED asthma cohort, and UK Biobank.[3] All cases (N=5135) were taking medication for asthma, and met the criteria for moderate-to-severe asthma according to the British Thoracic Society (BTS) 2014 guidelines. Controls (N=25,675) were from UK Biobank, and excluded those with a doctor-diagnosis of asthma, rhinitis, eczema, allergy, emphysema, or chronic bronchitis, or those with missing medication data. Analyses were adjusted for the first 10 principal components.

Overlap between the exposure and outcome datasets

Individuals from UK Biobank were included in the exposure blood cell GWAS and all outcome respiratory GWAS. There are varying degrees of sample overlap for each individual MR analysis.

UK Biobank initially released genetic data for up to ~150,000 participants (~50,000 genotyped on the UK BiLEVE array and selected according to extremes of lung function and smoking behaviour,[4] and an additional ~100,000 genotyped on the closely related UK Biobank Axiom array).[5] This is referred to as the 'interim release' of UK Biobank genetic data, and includes about 1/3 of UK Biobank

Guyatt A, *et al. Thorax* 2023; 78:496–503. doi: 10.1136/thoraxjnl-2021-217993

participants. The 'full release' of UK biobank genetic data followed later, and included data on >450,000 participants.

The sampling strategies for each GWAS were as follows: the exposure blood cell GWAS included up to ~173,000 individuals in total (exact sample size varied according to cell type, N=172,275 for eosinophils, see **Supplementary Table 3** for sample sizes of all cell types). This included up to 132,959 individuals from the UK Biobank first release of genetic data, and up to 40,521 samples from the INTERVAL study. Samples were of European ancestry.

The most prominent overlap was for the asthma GWAS dataset,[3] since individuals were also only sampled from the interim release of UK Biobank data (around 1/3 of participants). However, the asthma outcome GWAS was supplemented with cases from GASP and UBIOPRED.

The lung function, ACO and AECOPD GWAS data sets were sampled from 321,057 European ancestry individuals within the full release of UK Biobank genetic data who also had lung function measures passing QC.[2]

Whilst it is not possible to calculate the exact degree of sample overlap, likely estimates are presented overleaf, where overlap is the proportion of participants in the outcome GWAS who are also likely to feature in the exposure GWAS. For studies including UK Biobank data only, this figure is likely to be around 30%.

Guyatt A, *et al. Thorax* 2023; 78:496–503. doi: 10.1136/thoraxjnl-2021-217993

Estimation of % participants in outcome GWAS expected to feature in exposure GWAS

Exposure data set	Outcome data sets			% participants in outcome GWAS expected to feature in exposure GWAS*
Blood cell types	Outcome	UKB sample size and source (sampled from full release, or interim release of UKB genetic data)	Other studies sample size and source	
~132,959 participants sampled from interim release of UK Biobank genetic data<br><br>~40,521 INTERVAL participants[1]	Lung function (four traits)[2]	321,047 (full release)	79,055 (SpiroMeta)	23%
	Moderate-severe asthma[3]	2,996 cases (interim release)<br>25,600 controls (interim release)	1858+281 cases (GASP+UBIOPRED)<br>75 controls (UBIOPRED)	55% (cases)<br>94% (controls)
	ACO[6]	8,068 cases (full release)<br>40,360 controls (full release)		29% (cases)<br>29% (controls)
	Respiratory infections[7]	19,459 cases (full release)<br>101,438 controls (full release)		
	AECOPD	2,771 cases (full release)<br>42,052 controls (full release)		

*Core assumptions for calculations above:

- Assume phenotype availability is random with respect to genotype availability, for all GWAS
- 463,844 participants with genotype data and of European ancestry in full release[5]
- 152,725 genotyped participants in interim release[8]
- 141,751 of the above designated European ancestry in interim release[9]
- 132,959 (assumed as a subset of the above) in blood cell type GWAS (Astle et al. 2016)[1]

Statistical methods

Univariable MR analyses of eosinophils and all respiratory traits and diseases

As described in the main manuscript, we first performed MR analyses of eosinophils on all outcomes, including three quantitative lung function traits (FEV_1, FVC, and FEV_1/FVC); and four clinical disease phenotypes (asthma, AECOPD, ACO and respiratory infections), using genetic instrumental variables (IVs) selected from Astle *et al.* (2016)[1].

Astle *et al.* identified 209 conditionally distinct eosinophil count signals at $p<8.31x10^{-9}$ (their threshold for genome-wide significance), and we extracted effect sizes and standard errors for these SNPs from the meta-analysis of UKB and SpiroMeta from the GWAS of the three lung function traits.[2] Where SNPs were unavailable, we sought linkage disequilibrium (LD) proxies at $r^2>0.8$ in a European sample using the 'rAggr' tool (https://preventivemedicine.usc.edu/divisions/biostatistics/biostatistics-software/) that were also associated at $p<8.31x10^{-9}$ in the eosinophil GWAS.

Using the R package 'TwoSampleMR' (https://mrcieu.github.io/TwoSampleMR/, version 0.4.23), we harmonised the SNP-eosinophil and SNP-lung function associations so that the effect sizes corresponded to the same allele. For A/T and C/G SNPs, minor allele frequency (MAF) was used to infer strand, and SNPs were dropped from the analysis if they had a MAF of >0.42, since this precluded reliable strand inference. We then performed LD clumping to retain a set of 151 SNPs that were independent at $r^2<0.01$, using LD data from the European 1000 Genomes population. These SNPs (and proxies) were then additionally extracted from the other outcome GWAS (one SNP was missing from the asthma GWAS).

In the analyses of eosinophils and respiratory phenotypes, we report estimates from three MR methods, each of which are robust to different violations of the core assumptions shown in **Box 1**.

Inverse-variance weighted analyses

The primary analysis used the inverse-variance weighted (IVW) MR method, which combines Wald ratios (or for binary outcomes, Wald-type ratios[10]) of SNP-outcome to SNP-exposure effects across all SNPs by meta-analysis (we used a multiplicative random-effects model that corrects for under-dispersion in the model). The method requires that if SNPs are associated with the outcome via pathways other than the exposure (**Box 1**), the average effect through these pathways for these SNPs should be zero (e.g. any "horizontal pleiotropy" is 'balanced'). Moreover, horizontal pleiotropic effects should be unrelated to SNP-exposure effects (the "InSIDE" assumption—**In**strument **S**trength **I**ndependent of **D**irect **E**ffect).[11 12] We assessed horizontal pleiotropy by: i) computing Cochran's Q statistic to assess evidence of over-dispersion of causal estimates, ii) plotting SNP-exposure effects against SNP-outcome effects.[12]

MR-Egger analyses

MR-Egger analysis performs a weighted regression of SNP-outcome associations on SNP-exposure associations, allowing a non-zero intercept, so that potentially all IVs used could be invalid (e.g. have a non-zero effect on the outcome even when the effect of the exposure on the outcome is zero).[11] However, MR-Egger is sensitive to violation of the InSIDE assumption, and has less statistical power than the IVW and weighted median methods.

Weighted median analyses

The weighted median estimate is robust to violation of the InSIDE assumption and the presence of horizontal pleiotropy, provided that the IVs providing ≥50% of the total weight are valid, without having to specify which ones are invalid.[13]

MR-PRESSO analyses

We used MR-PRESSO to further assess for the possible impact of horizontal pleiotropy on our results. MR-PRESSO first conducts a 'global test', by comparing the observed residual sum of squares to the expected value, assuming no horizontal pleiotropy, for a group of variants. It then tries to identify specific variants which may be outliers due to horizontal pleiotropy, by comparing the observed and expected distributions of one variant only. A distortion test then quantifies the impact of removing the outliers on the causal estimate.[14]

Multivariable MR analyses of multiple blood cell types and respiratory outcomes

Instrument selection

Since SNPs affecting eosinophils also affect other blood count types,[1] we used multivariable MR in order to estimate the influence of multiple cell types on respiratory outcomes, after conditioning on the effects of the SNPs on other cell types (see below). Multivariable MR (MVMR) analyses were run for respiratory outcomes that showed evidence of eosinophil causation in the IVW MR analyses above, and that had broadly consistent effect estimates in the weighted median and MR-Egger analyses.

The aim of this analysis was therefore twofold: i) to further investigate the possibility of horizontal pleiotropy affecting the results of the eosinophil MR; and ii) to establish whether any other cell types besides eosinophils could affect the respiratory outcomes studied.

We extracted all of the genome-wide signals reported by Astle *et al.*[1] for GWAS of counts of the following blood cell types: eosinophils (as described), basophils, neutrophils, monocytes, lymphocytes, platelets, red blood cells, and reticulocytes. Across all traits, a total of 1166 SNPs were also available in the outcome GWAS.

We performed LD clumping across all 1166 SNPs (**Supplementary Table 3**). This resulting set of 318 SNPs (including SNPs associated with multiple traits) was then extracted from the GWAS results of each cell type, and also from each of the outcome GWAS. Harmonisation of SNP-exposure and SNP-outcome effects was as described previously.

Inverse-variance weighted MVMR

To implement inverse-variance weighted multivariable MR (IVW MVMR), we used the mv_multiple() function of the 'TwoSampleMR' R package.[12] This implements an approach described as a modification[15] to the MVMR method described by Burgess and Thompson.[16] Briefly, the method is performed by regressing the SNP-outcome associations on the SNP-exposure associations for all cell types simultaneously. This is therefore a multivariable weighted regression model (without an intercept), that uses inverse-variance weights.

Multivariable MR adjusting for weak instruments

MVMR estimation using the IVW approach (as above) is robust to the presence of balanced horizontal pleiotropy if the instrumental variables used are strong. To estimate the strength of the IVs in predicting each of the eight exposures, we calculated the conditional F-statistic for each exposure (**Supplementary Table 3**).[17] In the presence of weak instruments, false positive results for the detection of pleiotropy in MR analyses are more likely.

We calculated a modified form of Cochran's Q statistic (Q_A), described by Sanderson *et al*., this exact test for detecting pleiotropy in MVMR is robust even in the presence of weak instruments.[17] We implemented this using the pleiotropy_mvmr() function of the 'MVMR' R package.

We additionally used a method developed by the same authors to perform MVMR in the presence of moderately weak instruments. This approach estimates causal effects whilst accounting for excess heterogeneity (unrelated to variance in SNP-exposure or SNP-outcome associations) in the per-SNP effects, and is more robust to balanced pleiotropy. It was implemented using the qhet_mvmr() function in the 'MVMR' R package.

A jack-knife procedure was used to calculate standard errors ($\widehat{\mathrm{SE}}_{jack}$) for $\hat{\theta}$, the causal estimate, as adapted from [18]: briefly, each of $i = 1, 2, \ldots n$ SNP IVs was omitted in turn, and the causal estimate re-estimated for the ith jack-knife sample, giving n estimates in total, where $\hat{\theta}_{(i)}$ is the ith jack-knife replication of $\hat{\theta}$, e.g. the causal estimate from the dataset with the ith SNP IV removed. $\widehat{\mathrm{SE}}_{jack}$ for an exposure-outcome causal effect, $\hat{\theta}$, are then given as:

$$\widehat{\mathrm{SE}}_{jack} = [\frac{n-1}{n}\sum(\hat{\theta}_{(i)} - \hat{\theta}_{(.)})^2]^{1/2}$$

where $\hat{\theta}_{(.)} = \sum_{i=1}^{n} \hat{\theta}_{(i)}/n$

Multivariable MR, omitting variants contributing most to heterogeneity (quantified by Q statistic)

Finally, we examined the individual contribution of each SNP IV to the MVMR estimates, by omitting each SNP in turn. The absolute percentage reduction in the Q_A statistic after omitting a given SNP, compared to the Q_A statistic when including all SNPs in the model was calculated. SNPs that led to a reduction in Q by at least 2.5% were noted (**Supplementary Table 11**), and IVW MVMR models were recalculated without this subset of SNPs.

References

1. Astle WJ, Elding H, Jiang T, et al. The Allelic Landscape of Human Blood Cell Trait Variation and Links to Common Complex Disease. *Cell* 2016;167(5):1415-29 e19. doi: 10.1016/j.cell.2016.10.042 [published Online First: 2016/11/20]
2. Shrine N, Guyatt AL, Erzurumluoglu AM, et al. New genetic signals for lung function highlight pathways and chronic obstructive pulmonary disease associations across multiple ancestries. *Nat Genet* 2019;51(3):481-93. doi: 10.1038/s41588-018-0321-7 [published Online First: 2019/02/26]
3. Shrine N, Portelli MA, John C, et al. Moderate-to-severe asthma in individuals of European ancestry: a genome-wide association study. *Lancet Respir Med* 2019;7(1):20-34. doi: 10.1016/s2213-2600(18)30389-8 [published Online First: 2018/12/16]
4. Wain LV, Shrine N, Miller S, et al. Novel insights into the genetics of smoking behaviour, lung function, and chronic obstructive pulmonary disease (UK BiLEVE): a genetic association study in UK Biobank. *Lancet Respir Med* 2015;3(10):769-81. doi: 10.1016/s2213-2600(15)00283-0 [published Online First: 2015/10/02]
5. Bycroft C, Freeman C, Petkova D, et al. The UK Biobank resource with deep phenotyping and genomic data. *Nature* 2018;562(7726):203-09. doi: 10.1038/s41586-018-0579-z
6. John C, Guyatt AL, Shrine N, et al. Genetic associations and architecture of asthma-chronic obstructive pulmonary disease overlap. *medRxiv* 2020:2020.11.26.20236760. doi: 10.1101/2020.11.26.20236760
7. Williams A, Shrine N, Naghra-van Gijzel H, et al. Genome-wide association study of susceptibility to hospitalised respiratory infections [version 1; peer review: awaiting peer review]. *Wellcome Open Research* 2021;6(290) doi: 10.12688/wellcomeopenres.17230.1
8. Gudbjartsson DF, Bjornsdottir US, Halapi E, et al. Sequence variants affecting eosinophil numbers associate with asthma and myocardial infarction. *Nat Genet* 2009;41(3):342-7. doi: 10.1038/ng.323 [published Online First: 2009/02/10]

9. Shah RL, Guggenheim JA, Eye UKB, et al. Genome-wide association studies for corneal and refractive astigmatism in UK Biobank demonstrate a shared role for myopia susceptibility loci. *Human genetics* 2018;137(11-12):881-96. doi: 10.1007/s00439-018-1942-8 [published Online First: 2018/10/10]
10. Didelez V, Meng S, Sheehan NA. Assumptions of IV Methods for Observational Epidemiology. *Statist Sci* 2010;25(1):22-40. doi: 10.1214/09-STS316
11. Bowden J, Davey Smith G, Burgess S. Mendelian randomization with invalid instruments: effect estimation and bias detection through Egger regression. *Int J Epidemiol* 2015;44(2):512-25. doi: 10.1093/ije/dyv080 [published Online First: 2015/06/08]
12. Hemani G, Zheng J, Elsworth B, et al. The MR-Base platform supports systematic causal inference across the human phenome. *eLife* 2018;7:e34408. doi: 10.7554/eLife.34408
13. Bowden J, Davey Smith G, Haycock PC, et al. Consistent Estimation in Mendelian Randomization with Some Invalid Instruments Using a Weighted Median Estimator. *Genet Epidemiol* 2016;40(4):304-14. doi: 10.1002/gepi.21965 [published Online First: 2016/04/12]
14. Verbanck M, Chen C-Y, Neale B, et al. Detection of widespread horizontal pleiotropy in causal relationships inferred from Mendelian randomization between complex traits and diseases. *Nature Genetics* 2018;50(5):693-98. doi: 10.1038/s41588-018-0099-7
15. Burgess S, Dudbridge F, Thompson SG. Re: "Multivariable Mendelian Randomization: The Use of Pleiotropic Genetic Variants to Estimate Causal Effects". *American Journal of Epidemiology* 2015;181(4):290-91. doi: 10.1093/aje/kwv017
16. Burgess S, Thompson SG. Multivariable Mendelian randomization: the use of pleiotropic genetic variants to estimate causal effects. *Am J Epidemiol* 2015;181(4):251-60. doi: 10.1093/aje/kwu283 [published Online First: 2015/01/30]
17. Sanderson E, Spiller W, Bowden J. Testing and correcting for weak and pleiotropic instruments in two-sample multivariable Mendelian randomization. *Statistics in Medicine* 2021;40(25):5434-52. doi: https://doi.org/10.1002/sim.9133
18. Huang H. Jackknife-Bootstrap [PDF]. University of BerkeleyUnknown [cited 2021 21st December 2021]. Available from: https://www.stat.berkeley.edu/~hhuang/STAT152/Jackknife-Bootstrap.pdf accessed 21st December 2021.
