# Supplementary material for: Mendelian randomisation of eosinophils and other cell types in relation to lung function and disease
Source: Thorax. 2022 May 10;78(5):496–503. doi: 10.1136/thoraxjnl-2021-217993 (PMC10176352; doi:10.1136/thoraxjnl-2021-217993)
Supplement: Supplementary data [file thoraxjnl-2021-217993supp002.pdf]

Supplementary Figure 1

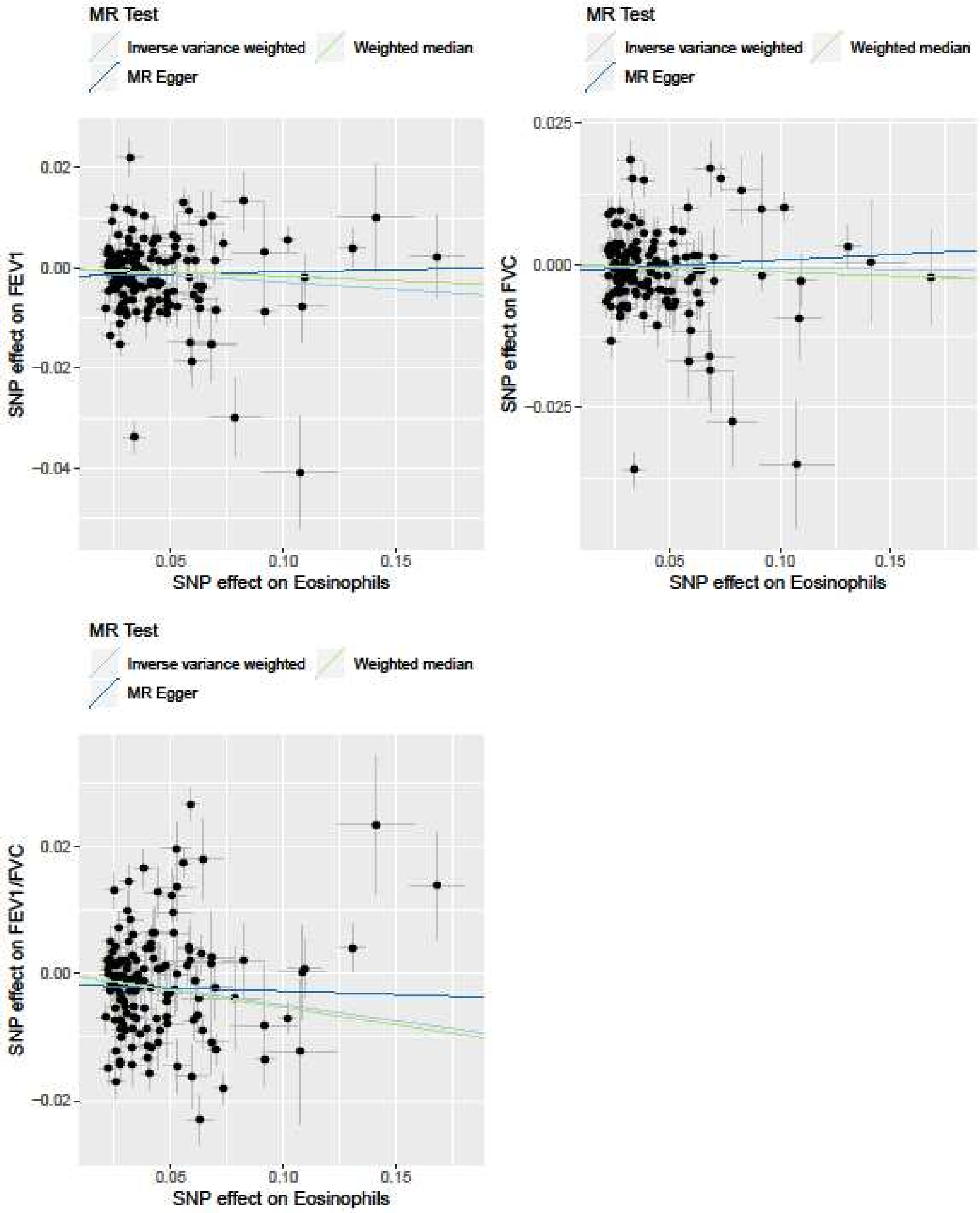

*These scatterplots show the relationship between the SNP-eosinophil and SNP-outcome effects for 151 SNPs used in univariable MR analyses, for three quantitative lung function traits: forced expiratory volume in 1 second (FEV_1), forced vital capacity, and the ratio of FEV_1/FVC. Each black dot represents a SNP, and whiskers represent precision. The three fitted lines correspond to the causal effect estimates using three separate MR methods. Units are in SD change (see **Extended Methods**).*

Supplementary Figure 2

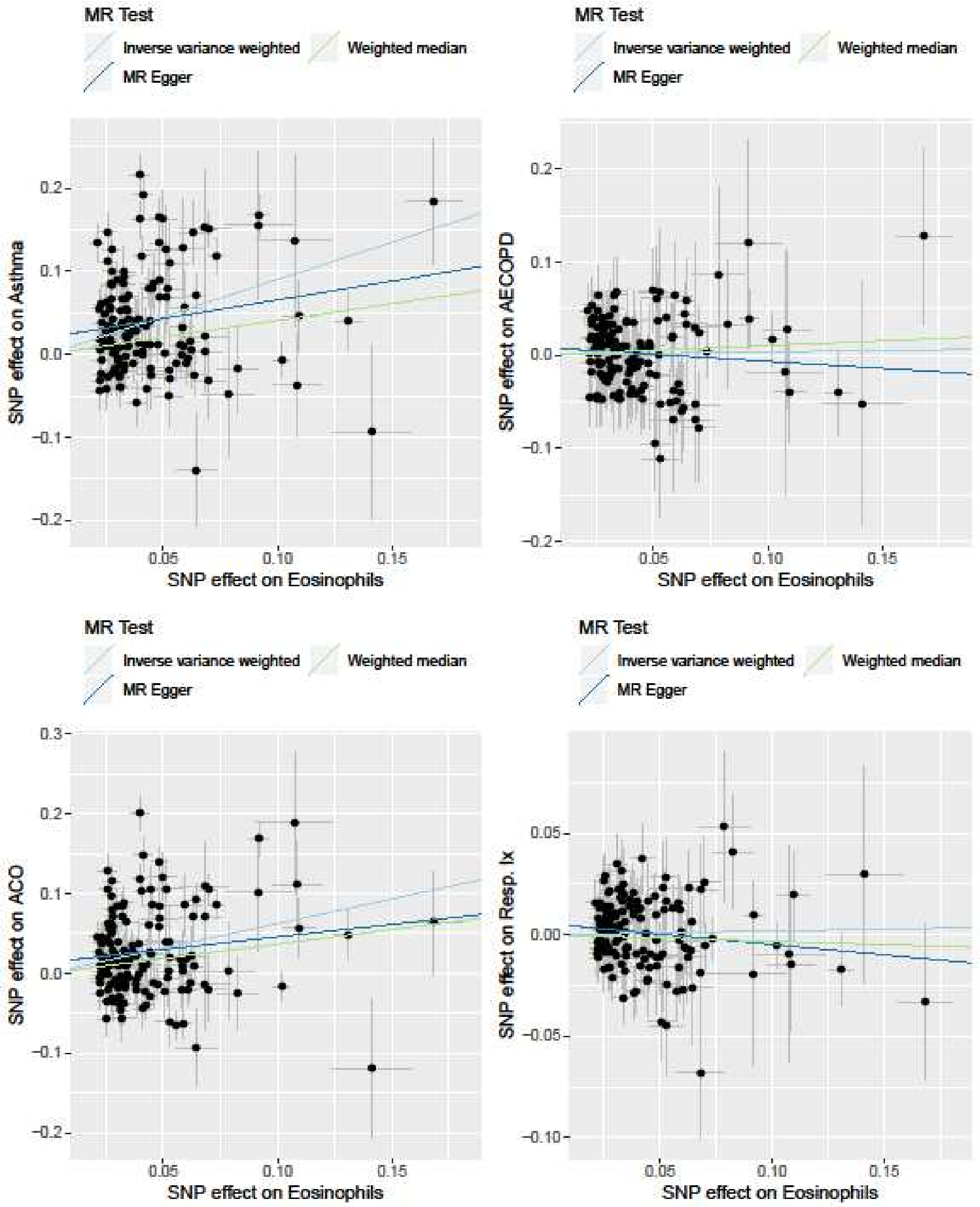

These scatterplots show the relationship between the SNP-eosinophil and SNP-outcome effects for 151 SNPs used in univariable MR analyses, for four binary respiratory phenotypes: moderate-to-severe asthma, acute exacerbations of COPD, asthma-COPD overlap, and respiratory infections. Each black dot represents a SNP, and whiskers represent precision. The three fitted lines correspond to the causal effect estimates using three separate MR methods. Units are in SD change (see ***Extended Methods****).*

Supplementary Figure 3

Trait	Outcome	Beta_95CI	P
Basophils	FEV1FVC	−0.002 (−0.06, 0.056)	0.939
	FEV1FVC (UKB)	−0.005 (−0.069, 0.058)	0.876
	FEV1FVC (UKB asthma)	−0.025 (−0.133, 0.083)	0.648
	FEV1FVC (UKB no asthma)	−0.006 (−0.068, 0.055)	0.839
Eosinophils	FEV1FVC	−0.065 (−0.104, −0.026)	0.001
	FEV1FVC (UKB)	−0.071 (−0.113, −0.028)	0.001
	FEV1FVC (UKB asthma)	−0.093 (−0.165, −0.021)	0.011
	FEV1FVC (UKB no asthma)	−0.028 (−0.069, 0.013)	0.176
Lymphocytes	FEV1FVC	0.032 (−0.008, 0.071)	0.117
	FEV1FVC (UKB)	0.044 (0.001, 0.087)	0.047
	FEV1FVC (UKB asthma)	0.081 (0.008, 0.154)	0.03
	FEV1FVC (UKB no asthma)	0.032 (−0.01, 0.074)	0.133
Monocytes	FEV1FVC	0.013 (−0.014, 0.04)	0.34
	FEV1FVC (UKB)	0.015 (−0.014, 0.044)	0.319
	FEV1FVC (UKB asthma)	0.006 (−0.043, 0.055)	0.82
	FEV1FVC (UKB no asthma)	0.009 (−0.019, 0.037)	0.515
Neutrophils	FEV1FVC	−0.002 (−0.04, 0.035)	0.901
	FEV1FVC (UKB)	−0.004 (−0.046, 0.037)	0.834
	FEV1FVC (UKB asthma)	−0.027 (−0.097, 0.043)	0.456
	FEV1FVC (UKB no asthma)	0.009 (−0.031, 0.049)	0.669
Platelets	FEV1FVC	0.003 (−0.021, 0.027)	0.798
	FEV1FVC (UKB)	0 (−0.026, 0.027)	0.98
	FEV1FVC (UKB asthma)	−0.009 (−0.053, 0.036)	0.706
	FEV1FVC (UKB no asthma)	−0.001 (−0.027, 0.024)	0.926
RBCs	FEV1FVC	−0.019 (−0.049, 0.011)	0.22
	FEV1FVC (UKB)	−0.019 (−0.052, 0.014)	0.255
	FEV1FVC (UKB asthma)	−0.034 (−0.089, 0.021)	0.229
	FEV1FVC (UKB no asthma)	−0.018 (−0.05, 0.013)	0.258
Retics	FEV1FVC	0.002 (−0.023, 0.028)	0.871
	FEV1FVC (UKB)	0.001 (−0.027, 0.029)	0.959
	FEV1FVC (UKB asthma)	−0.022 (−0.07, 0.025)	0.356
	FEV1FVC (UKB no asthma)	0 (−0.027, 0.026)	0.973

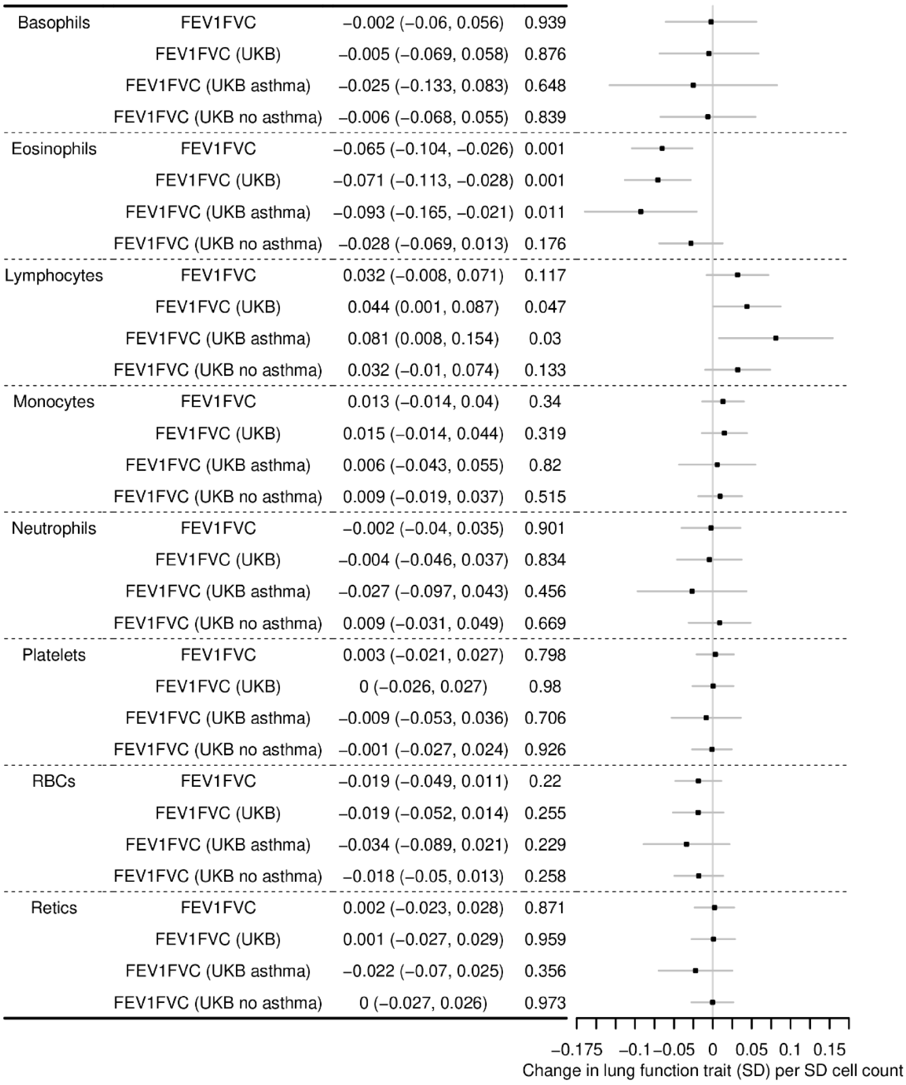

Legend

Forest plot showing multivariable MR estimating the causal effect of multiple cell types on the ratio of forced expiratory volume in 1 second (FEV_1) to forced vital capacity (FVC), after conditioning on the effects of the SNPs on other cell types. Models were run using four sets of SNP-outcome results: GWAS results for FEV_1/FVC from UK Biobank and SpiroMeta [FEV1FVC, N=400,102]; results from UK Biobank only [FEV1FVC (UKB), N=321,047];

results from UK Biobank only, restricting to individuals with asthma [FEV1FVC (UKB asthma), N=37,868]; and results from UK Biobank only, after excluding individuals with asthma [FEV1FVC (UKB no asthma), N=283,179]. Effect sizes (beta, 95% confidence interval, 95CI) are in SD change in lung function outcome per SD cell count (adjusted for the effects of other cell types). Points of the forest plot represent effect size estimate; whiskers are 95% confidence intervals.
